# Composite dietary antioxidant index in relation to urge urinary incontinence in US men

**DOI:** 10.3389/fnut.2024.1514320

**Published:** 2024-12-20

**Authors:** Xuefeng Jin, Wenhui Tong, Li Sun, Sujue Lu, Tiantian Xu, Pan Sun, Yan Liu, Hangxu Li

**Affiliations:** ^1^Medical College of Jinzhou Medical University, Jinzhou, Liaoning, China; ^2^Medical College of Yangzhou University, Yangzhou, Jiangsu, China; ^3^Medical College of Nantong University, Nantong, Jiangsu, China; ^4^Medical College of Shaanxi University of Chinese Medicine, Xianyang, Shanxi, China; ^5^Department of Urology, The First Affiliated Hospital of Jinzhou Medical University, Jinzhou, China; ^6^Department of Urology, The Third Affiliated Hospital of Jinzhou Medical University, Jinzhou, Liaoning, China

**Keywords:** NHANES, UUI (urge urinary incontinence), composite dietary antioxidant index (CDAI), propensity score matching (PSM), exogenous antioxidants

## Abstract

**Background:**

Urinary incontinence (UI), particularly urge urinary incontinence (UUI), is a prevalent condition that worsens with age and negatively affects quality of life. Antioxidants, measured by the composite dietary antioxidant index (CDAI), have been linked to inflammation and other diseases, but their relationship with UUI remains uncertain. The purpose of this study is to investigate the relationship between UUI prevalence and CDAI.

**Materials and methods:**

Data for this cross-sectional study were obtained from the National Health and Nutrition Examination Survey’s four cycles (2011–2018). The odds ratio (OR) and 95% confidence interval (95% CI) of the relationship between CDAI and male UUI were ascertained by the use of weighted univariate analysis, multivariate logistic regression, restricted cubic spline regression, and subgroup analysis. PSM and sensitivity analyses were performed to assess the robustness of the findings.

**Results:**

A total of 7,735 participants took part in this study. After adjusting for potential confounders, CDAI was found to be negatively associated with the prevalence of UUI in those with lower CDAI (about half overall). This relationship lost significance in populations with higher CDAI. The negative correlation between zinc and the prevalence of UUI was more significant in populations with low antioxidant diets. The results remained consistent, with subgroup analyses finding a significant interaction effect for race only after PSM (*p* = 0.043), with no significant interaction effect observed for the rest.

**Conclusion:**

This study showed a negative correlation between CDAI and UUI incidence in the group of men with low CDAI levels (about half of the population). Thus, effective prevention or treatment of UUI requires dietary changes aimed at the male population with poor antioxidant diets.

## 1 Introduction

Urinary incontinence (UI) is a common disorder in the population, usually increasing in prevalence with age (1). The unintentional loss of pee during urine storage is classified as urinary incontinence (2). Senior age, High BMI, menopause, and childbearing have all been linked to an increased risk of UI, according to previous research (3–6). Stress UI (SUI), Urge UI (UUI), and mixed UI (MUI) are the three most prevalent forms of UI (7). The ageing of the world’s population is making UI a serious public health issue.

The sudden, overwhelming urge to urinate followed right away by involuntary incontinence is known as urge urinary incontinence (UUI) (8). According to estimates, UUI affects a significant percentage of people in the US population—2.6 to 20.9% of males and 9.3 to 30.8% of women—and its incidence rises sharply with age (9, 10). UUI can be a debilitating illness that causes significant deficits in self-confidence and psychological well-being in addition to a reduction in social contacts and interpersonal connections (11). These symptoms have a substantial negative influence on the quality of life and frequently call for behavioural, pharmaceutical, or surgical therapies.

Diet as a part of behavioural therapy is now being considered a promising treatment for many diseases (12–15). The composite dietary antioxidant index (CDAI) is based on a range of dietary vitamins and minerals with antioxidant properties, including carotenoids, zinc, selenium, and vitamins C, E, and A. This summary score is used to assess a person’s dietary total antioxidant capacity (TAC) (16, 17). Recent research indicates a link between CDAI and inflammatory biomarkers (18). It is widely recognized by scholars that considering a composite diet is more representative of actual daily nutrient intake in humans, rather than focusing on individual nutrients (19). Therefore, this study explores the relationship between the combination of the six trace nutrients mentioned above and UUI.

Numerous investigations have now demonstrated the connection between illnesses and CDAI (20–24). It’s still unclear, though, how CDAI and UUI are related. Previous studies have focused on the relationship between inflammation-related indices and female UUI, but there is relatively little research on inflammation and UUI in men. The National Health and Nutrition Examination Survey (NHANES) 2011–2018 data are used in this cross-sectional study to investigate any possible relationship between CDAI (and its components) and UUI prevalence in men. We also examined the potential pathways, and our findings should serve as a foundation for an exogenous antioxidant diet that prevents UUI.

## 2 Materials and methods

### 2.1 Information sources consulted for this study

One of the most important programs of the National Center for Health Statistics (NCHS) is the NHANES. Data that is indicative of the nation on the general health of the US population has been collected (25). With the use of a sophisticated multistage probability methodology, this program produces a nationally representative sample of non-institutionalized Americans (26). Since 1999, it has used a sophisticated, stratified, multi-stage probability sampling method to gather data from about five thousand people each year (27). IN addition, its survey cycle lasts 2 years. All procedures were approved by the NCHS Research Ethics Committee, and each participant gave their informed permission (28). Data from questionnaires, laboratory test indicators, physical examination characteristics, and sociodemographic status were also collected. There are extensive details about the program on the website.^1^

### 2.2 Study population in this investigation

**Figure 1:**
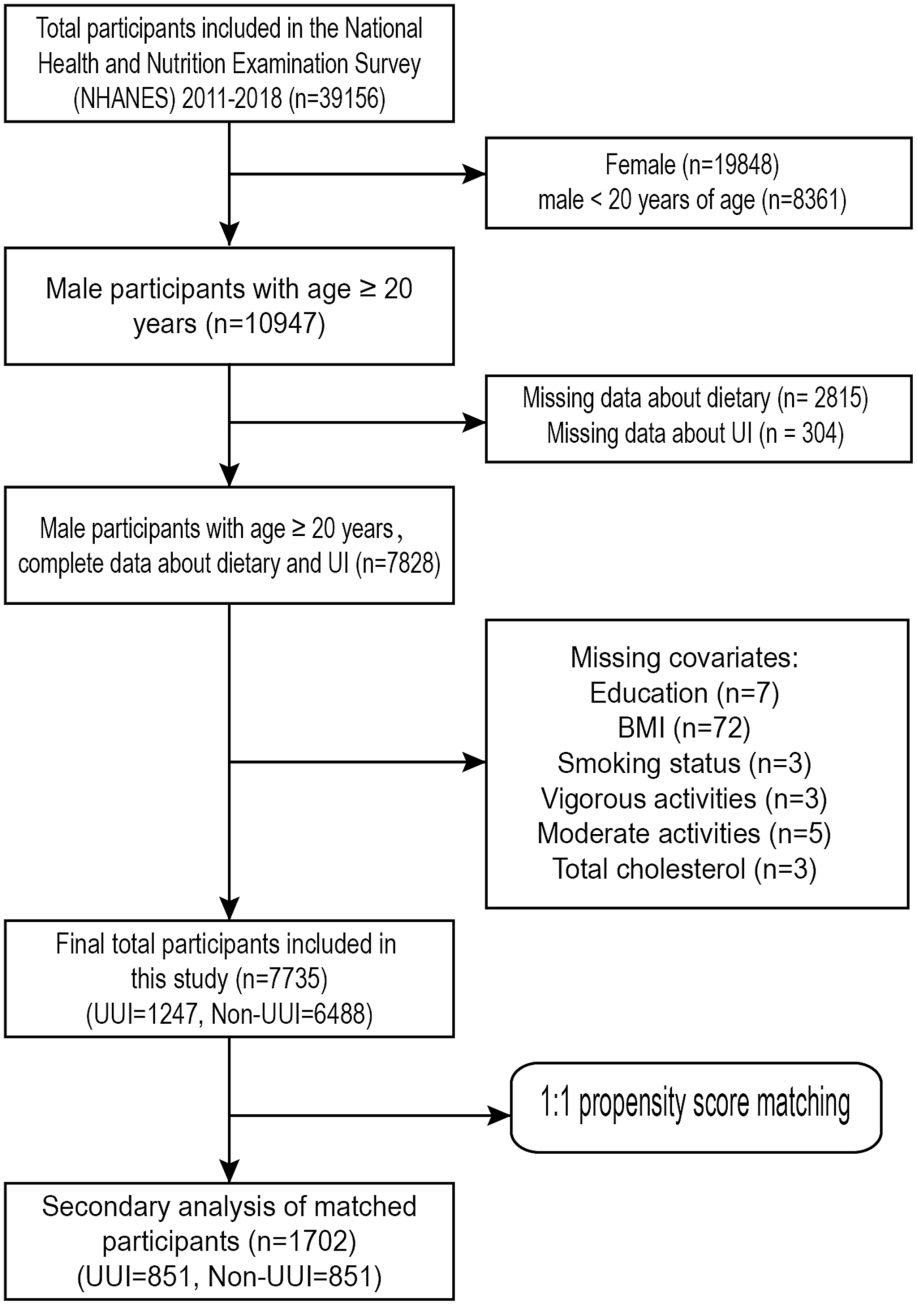
Flowchart for screening research participants.

This study analysed data from four NHANES cycles, spanning from 2011 to 2018. The survey was completed by 39,156 people in total (Representing an actual population of 103,877,855). Firstly, we excluded females (*n* = 19,848) and males under 20 years of age (*n* = 8,361). The subsequent exclusion criteria were as follows: (1) information about dietary (*n* = 2,815) is unknown; (2) interviewees who had not finished the UI survey (*n* = 304); (3) data about education status (*n* = 7) is unknown; (4) body mass index (*n* = 72) is unknown; (5) data about smoking status (*n* = 3) is unknown; (6) information about vigorous activities (*n* = 3) is unknown; (7) information about moderate activities (*n* = 5) is unknown; and (8) Total cholesterol (*n* = 3) is unknown. Finally, the study comprised 7,735 individuals in total after screening procedures, including 1,247 men with UUI and the remaining without UUI. Figure 1 shows the screening procedures.

### 2.3 Measurement of CDAI

The 24 h dietary recall interview is the current section of the NHANES nutritional evaluation. Dietary interviewers with training who are fluent in both Spanish and English perform dietary recall interviews in person. Through discontinuous two-day, 24 h dietary recall interviews, the NHANES gathered data on participants’ food intake. A mobile examination center (MEC) served as the venue for the initial interview. Every mobile examination center had a nutritional interview room with a common set of measurement guidelines. The second dietary recall interview took place over the phone three to ten days later (29, 30).

We used the average of two measurements to minimize bias and increase the reliability of the results. A modified version developed by previous researchers was utilized to calculate each participant’s CDAI (31). Zinc, selenium, carotenoids, and vitamins A, C, and E are the six dietary antioxidants that make up CDAI. We standardized each micronutrient by subtracting the mean of the six dietary vitamins and minerals and dividing by the total standard deviation to calculate a Z-score. Their Z-scores were then summed to obtain the composite value of CDAI. The following is the calculating formula:


$$\mathrm{CDAI}=\overset{n=6}{\underset{i=1}{\sum }}\left(\mathrm{Individual}\;\mathrm{Intake}-\mathrm{Mean}\right)/SD$$

### 2.4 Evaluation of UI

In MECs (Mobile Examination Centres), only those who were ≥ 20 years old answered questions on urine incontinence. “During the past 12 months, have you leaked or lost control of even a small amount of urine with activity like coughing, lifting, or exercise?” asked participants, if they selected “yes.” grouped under “stress UI.” A positive response to the question, “During the past 12 months, have you leaked or lost control of even a small amount of urine with an urge or pressure to urinate and you could not get to the toilet fast enough?” formed the basis for the definition of “urgency UI.” If a participant answered “yes” to both the stress and urgency UI questions, they were categorized as mixed UI participants.

### 2.5 Evaluation of covariate

The following potential confounding variables were selected for this investigation based on prior research findings that may affect UI results. These variables included demographics, medical history, physical examination findings, and personal circumstances (6, 8, 32–35). Race, age, education, PIR, smoking status, alcohol use, diabetes, hypertension, total cholesterol levels, and exercise status were all considered categorical factors.

We then grouped these covariates. Two groups were formed from the participants according to their age, which was fifty. The age range of the first group was under 50, whereas the age range of the second group was over or equal to 50. Three categories—below high school diploma, high school diploma, and above high school diploma—were used to classify people’s educational positions. PIR < 2 was classified as low PIR, and PIR ≥ 2 as high PIR. We divided BMI into three groups: the first group was less than 25, the second group was greater than or equal to 25 and less than 30, and the third group was less than 30. When asked if they smoked, participants were divided as smokers if they replied “yes” (SMQ020). Answer the query “In the past 12 months, on those days that you drank alcoholic beverages, on average, how many drinks did you have?” to distinguish between drinkers and non-drinkers. Those who had less than 12 drinks were categorized as non-drinkers, while those who had more than or equal to 12 drinks were considered drinkers.

Those who responded “yes” to the inquiry “Have you been told you have hypertension?,” those who were taking antihypertensive medication, and those whose average of three measurements of systolic blood pressure (BP) was ≥140 mmHg and whose average of three measurements of diastolic blood pressure (BP) was ≥90 mmHg were all considered patients with hypertension. Participants were considered to have diabetes if they answered “yes” when asked if they had diabetes or if they used glucose-lowering drugs or insulin. Meanwhile, their fasting blood glucose (≥126 mg/dL) and glycosylated haemoglobin (≥6.5%) were used to diagnose diabetes. Total cholesterol <240 mg/dL was defined as a low cholesterol level and ≥ 240 mg/dL was defined as a high cholesterol level.

### 2.6 Statistical analysis

During the processing phase, NHANES sample weights were applied to guarantee the study population’s national representation. The baseline qualities were explained after the participants were classified according to the four CDAI categories. Categorical variables, expressed as weighted percentages (%), were compared using a chi-square test. Continuous variables were compared using weighted linear regression, and the results were shown as mean (± standard deviation).

To reduce confounding bias, we performed PSM processing based on UUI results. To ensure that the distribution of covariates was comparable between the UUI and non-UUI groups, we matched for the following factors: age, smoking, alcohol consumption, diabetes, hypertension, and total cholesterol (36–40). Propensity score matching (PSM) was carried out 1:1 using the R Software “MatchIt” program. The sample population was re-examined after PSM in order to validate the findings.

To determine whether there were any noteworthy trends or differences, a univariate analysis was performed. The study then developed three adjustment models in order to use multivariate regression analysis to examine the relationships between CDAI and UUI. Model 1 was the baseline model that did not alter any covariate variables. Race, age, education, and PIR were added to model 2. Model 3 included BMI, diabetes, hypertension, smoking, alcohol consumption, total cholesterol level, moderate activity, and vigorous activity on the basis of Model 2. A generalized additive model (GAM) was then developed to validate the dose–response relationship. A threshold effect analysis was first performed. Then, restricted cubic spline (RCS) and smooth curve fitting were used to describe the dose–response relationship between CDAI and UUI. The study used RCS to investigate whether there was a nonlinear association between CDAI and UUI, with the node set to 3. Smooth curve fitting was performed under the fully modified model. After obtaining the threshold value, the distribution of CDAI in the population was made into a violin plot for the next analysis.

We then categorized the population according to the thresholds and then regressed the CDAI and its components on the UUI separately to explore whether certain elements had a greater effect. Then, using subgroup analysis, the stratified association between CDAI and UUI was examined. Additionally, interaction tests were performed to assess the way in which relationships between subgroups interacted.

For all statistical studies, R (version 4.4.0) was utilized. When the two-sided *p*-value was less than 0.05, it was deemed statistically significant.

## 3 Results

### 3.1 Population characteristics

Based on the screening criteria, a total of 7,735 eligible participants from NHANES 2011–2018 were selected (Figure 1). Among them, 1,247 had UUI and 6,488 did not. The weighted estimates for the baseline characteristics of the study population are shown in Table 1. Study participants with a higher CDAI were found to be more heavily represented under the age of 50. In addition, those with higher levels of CDAI were more likely to be non-Hispanic white, more educated, have a higher PIR, lower smoking rates, lower alcohol consumption rates, lower body mass index, moderate exercise intensity, lower probability of having a history of diabetes and hypertension, and lower total cholesterol levels than those with lower levels of CDAI.

**Table 1:** Weighted basic characteristics of screened participants (*N* = 7,735) before PSM.

Characteristics	Total (*n* = 7,735)	CDAI				*p*-value
		1 (*n* = 1934)	2 (*n* = 1933)	3 (*n* = 1934)	4 (*n* = 1934)	
Age, years						<0.001
<50	3,771 (48.75)	859 (44.42)	899 (46.51)	938 (48.50)	1,075 (55.58)	
≥50	3,964 (51.25)	1,075 (55.58)	1,034 (53.49)	996 (51.50)	859 (44.42)	
Race						<0.001
Mexican American	989 (12.79)	212 (10.96)	267 (13.81)	265 (13.70)	245 (12.67)	
Other Hispanic	733 (9.48)	205 (10.60)	176 (9.11)	178 (9.20)	174 (9.00)	
Non-Hispanic white	3,087 (39.91)	689 (35.63)	762 (39.42)	821 (42.45)	815 (42.14)	
Non-Hispanic black	1739 (22.48)	573 (29.63)	424 (21.93)	366 (18.92)	376 (19.44)	
Other	1,187 (15.35)	255 (13.19)	304 (15.73)	304 (15.72)	324 (16.75)	
Education						<0.001
Below high school level	1,559 (20.16)	554 (28.65)	391 (20.23)	325 (16.80)	289 (14.94)	
High school diploma	1820 (23.53)	516 (26.68)	467 (24.16)	408 (21.10)	429 (22.18)	
More than high school	4,356 (56.32)	864 (44.67)	1,075 (55.61)	1,201 (62.10)	1,216 (62.87)	
PIR						<0.001
<2	3,191 (45.01)	975 (55.49)	790 (44.81)	684 (38.62)	742 (41.25)	
≥2	3,899 (54.99)	782 (44.51)	973 (55.19)	1,087 (61.38)	1,057 (58.75)	
BMI (kg/m^2^)						<0.001
<25	2073 (26.80)	528 (27.30)	480 (24.83)	483 (24.97)	582 (30.09)	
25–29.99	2,851 (36.86)	683 (35.32)	721 (37.30)	737 (38.11)	710 (36.71)	
≥30	2,811 (36.34)	723 (37.38)	732 (37.87)	714 (36.92)	642 (33.20)	
Smoking						<0.001
Yes	4,044 (52.28)	1,120 (57.91)	1,010 (52.25)	977 (50.52)	937 (48.45)	
No	3,691 (47.72)	814 (42.09)	923 (47.75)	957 (49.48)	997 (51.55)	
Alcohol consumption						0.048
No	5,501 (96.90)	1,290 (95.84)	1,373 (97.10)	1,408 (97.64)	1,430 (96.95)	
Yes	176 (3.10)	56 (4.16)	41 (2.90)	34 (2.36)	45 (3.05)	
Hypertension						<0.001
No	4,266 (55.15)	978 (50.57)	1,045 (54.06)	1,084 (56.05)	1,159 (59.93)	
Yes	3,469 (44.85)	956 (49.43)	888 (45.94)	850 (43.95)	775 (40.07)	
Diabetes						<0.001
No	6,187 (79.99)	1,493 (77.20)	1,525 (78.89)	1,551 (80.20)	1,618 (83.66)	
Yes	1,548 (20.01)	441 (22.80)	408 (21.11)	383 (19.80)	316 (16.34)	
Vigorous activity						0.216
No	5,571 (72.02)	1,426 (73.73)	1,393 (72.06)	1,382 (71.46)	1,370 (70.84)	
Yes	2,164 (27.98)	508 (26.27)	540 (27.94)	552 (28.54)	564 (29.16)	
Moderate activity						0.002
No	4,438 (57.38)	1,171 (60.55)	1,116 (57.73)	1,057 (54.65)	1,094 (56.57)	
Yes	3,297 (42.62)	763 (39.45)	817 (42.27)	877 (45.35)	840 (43.43)	
Total cholesterol						0.002
Low level	4,370 (56.50)	1,109 (57.34)	1,055 (54.58)	1,053 (54.45)	1,153 (59.62)	
High level	3,365 (43.50)	825 (42.66)	878 (45.42)	881 (45.55)	781 (40.38)	
SUI						0.988
No	7,343 (94.93)	1835 (94.88)	1834 (94.88)	1835 (94.88)	1839 (95.09)	
Yes	392 (5.07)	99 (5.12)	99 (5.12)	99 (5.12)	95 (4.91)	
UUI						<0.001
No	6,488 (83.88)	1,578 (81.59)	1,640 (84.84)	1,606 (83.04)	1,664 (86.04)	
Yes	1,247 (16.12)	356 (18.41)	293 (15.16)	328 (16.96)	270 (13.96)	
MUI						0.56
No	7,506 (97.04)	1873 (96.85)	1881 (97.31)	1870 (96.69)	1882 (97.31)	
Yes	229 (2.96)	61 (3.15)	52 (2.69)	64 (3.31)	52 (2.69)	

Data are shown as *n* (%). OR, odds ratio; CI, confidence interval.

We found that differences in CDAI levels differed significantly in the presence or absence of UUI and that participants with UUI had lower CDAI levels. However, no significant CDAI level differences were observed in SUI and MUI.

**Figure 2:**
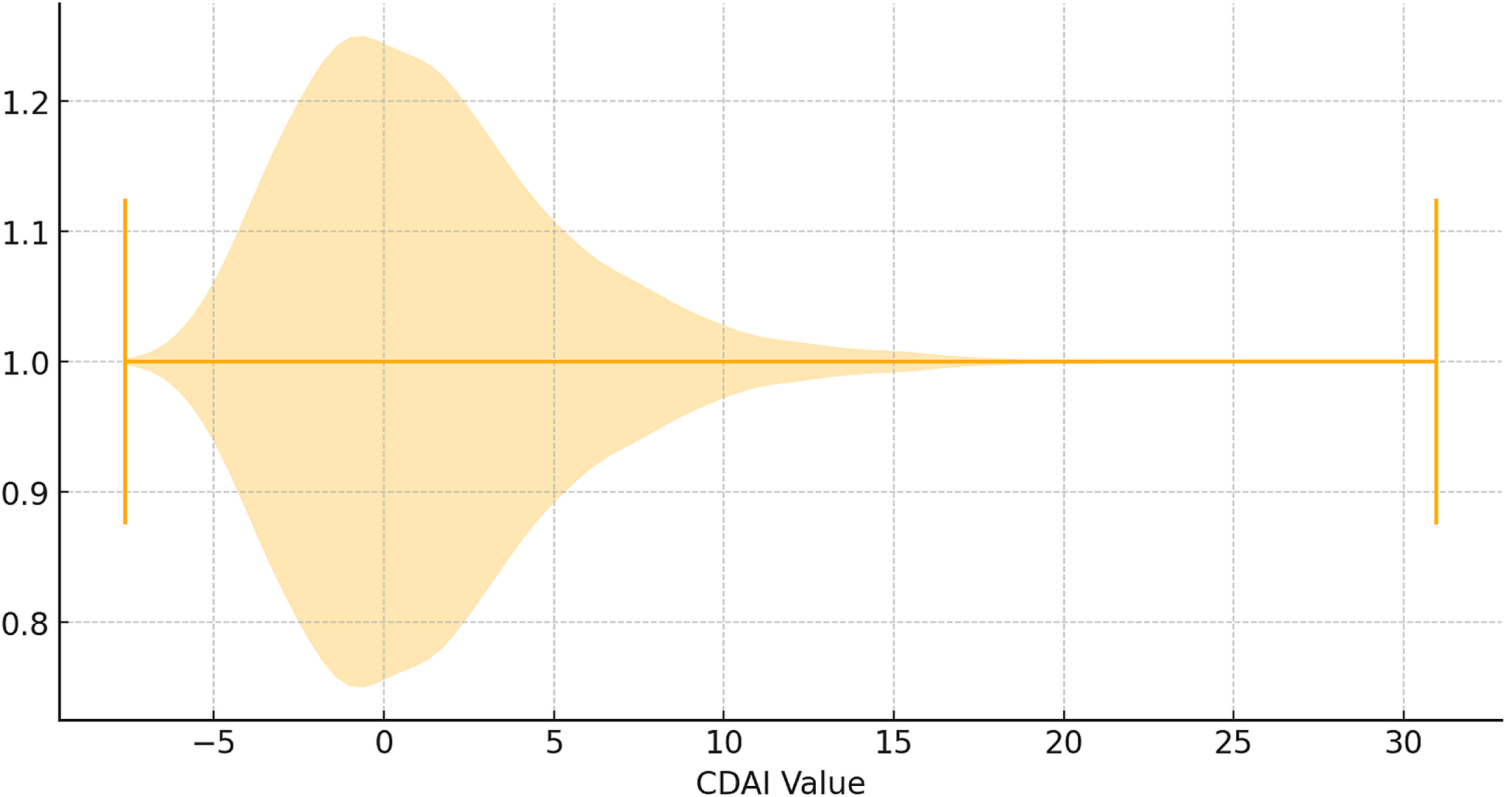
Violin plot of CDAI values.

Afterwards, the CDAI distribution was analysed and visualized as a violin plot (Figure 2). Many people have CDAI values clustered in the lower range, even less than 0 in a large proportion, while the number of people with higher antioxidant indices is smaller (median CDAI: 0.776).

We then balanced the effect of potential confounders associated with UUI through propensity score matching (PSM) analysis. In this investigation, a 1:1 PSM analysis was conducted. The standardized mean difference is visualized in Supplementary Figure S1, and the distributional balance plot of PSM is shown in Supplementary Figure S2. Following PSM, 1702 participants were enrolled in the study; 851 of them had UUI, while the remaining individuals did not (Figure 1). The weighted essential attributes of the research subjects were displayed in Supplementary Table S1 following PSM. Differences in variables between the two groups were managed to some degree. After PSM, age, race, education, PIR, hypertension, total cholesterol, vigorous activity, and UUI were found to be significantly associated with CDAI.

### 3.2 Univariate analysis of UUI

Preliminary exploration of pre- and post-PSM data with a univariate analysis of UUI. We found the following: prior to PSM, UUI was positively associated with age ≥ 50 years, body mass index ≥25, non-Hispanic black, hypertension, diabetes mellitus, and high cholesterol. In addition, UUI was negatively associated with other races, high school and higher education, high PIR, nonsmokers, vigorous activity, and Q2 and Q4 in the CDAI quartiles. After PSM, UUI was not associated with covariates such as age, high school education, 25 ≤ BMI < 30, smoking, hypertension, diabetes mellitus, level of exercise, and cholesterol level.

After PSM, only BMI ≥ 30 was positively associated with UUI. In contrast, other races, higher than high school education level, high PIR, and Q2 in CDAI were negatively associated with UUI (Table 2). We found that Q2 in CDAI was negatively correlated with UUI both before and after PSM (*p* = 0.007 and *p* = 0.004).

**Table 2:** Univariate regression analysis before and following PSM.

Variables	Before PSM		After PSM	
	OR (95%CI)	*p*	OR (95%CI)	*p*
Age (years)				
<50	1.00 (Reference)		1.00 (Reference)	
≥50	6.12 (5.23 ~ 7.16)	<0.001	1.00 (0.79 ~ 1.27)	1.000
Race				
Mexican American	1.00 (Reference)		1.00 (Reference)	
Other Hispanic	0.95 (0.73 ~ 1.25)	0.732	1.00 (0.66 ~ 1.52)	0.988
Non-Hispanic white	1.06 (0.87 ~ 1.29)	0.588	0.93 (0.68 ~ 1.27)	0.642
Non-Hispanic black	1.57 (1.28 ~ 1.93)	<0.001	1.39 (1.00 ~ 1.92)	0.052
Other	0.48 (0.37 ~ 0.63)	<0.001	0.55 (0.37 ~ 0.82)	0.003
Education				
Below high school level	1.00 (Reference)		1.00 (Reference)	
High school diploma	0.80 (0.68 ~ 0.96)	0.014	0.81 (0.61 ~ 1.08)	0.147
More than high school	0.64 (0.55 ~ 0.74)	<0.001	0.67 (0.52 ~ 0.86)	0.001
PIR				
<2	1.00 (Reference)		1.00 (Reference)	
≥2	0.81 (0.71 ~ 0.92)	<0.001	0.82 (0.68 ~ 0.99)	0.037
BMI (kg/m^2^)				
<25	1.00 (Reference)		1.00 (Reference)	
25–29.99	1.20 (1.02 ~ 1.42)	0.027	1.05 (0.81 ~ 1.35)	0.712
≥30	1.60 (1.36 ~ 1.87)	<0.001	1.28 (1.01 ~ 1.65)	0.049
Smoking				
Yes	1.00 (Reference)		1.00 (Reference)	
No	0.57 (0.50 ~ 0.65)	<0.001	1.01 (0.82 ~ 1.23)	0.959
Alcohol consumption				
No	1.00 (Reference)		1.00 (Reference)	
Yes	0.81 (0.51 ~ 1.27)	0.348	1.05 (0.57 ~ 1.92)	0.877
Hypertension				
No	1.00 (Reference)		1.00 (Reference)	
Yes	2.77 (2.44 ~ 3.15)	<0.001	1.01 (0.83 ~ 1.23)	0.920
Diabetes				
No	1.00 (Reference)		1.00 (Reference)	
Yes	2.71 (2.37 ~ 3.09)	<0.001	0.99 (0.81 ~ 1.22)	0.959
Vigorous activity				
No	1.00 (Reference)		1.00 (Reference)	
Yes	0.74 (0.64 ~ 0.85)	<0.001	0.91 (0.73 ~ 1.13)	0.405
Moderate activity				
No	1.00 (Reference)		1.00 (Reference)	
Yes	0.91 (0.80 ~ 1.03)	0.125	0.99 (0.82 ~ 1.20)	0.922
Total cholesterol				
Low level	1.00 (Reference)		1.00 (Reference)	
High level	2.17 (1.91 ~ 2.45)	<0.001	1.00 (0.82 ~ 1.21)	1.000
CDAI quantile				
Q1	1.00 (Reference)		1.00 (Reference)	
Q2	0.79 (0.67 ~ 0.94)	0.007	0.68 (0.52 ~ 0.89)	0.004
Q3	0.91 (0.77 ~ 1.07)	0.238	0.89 (0.68 ~ 1.16)	0.392
Q4	0.72 (0.61 ~ 0.85)	<0.001	0.84 (0.65 ~ 1.10)	0.217

OR, odds ratio; CI, confidence interval.

### 3.3 The relationships between CDAI and UUI

**Figure 3:**
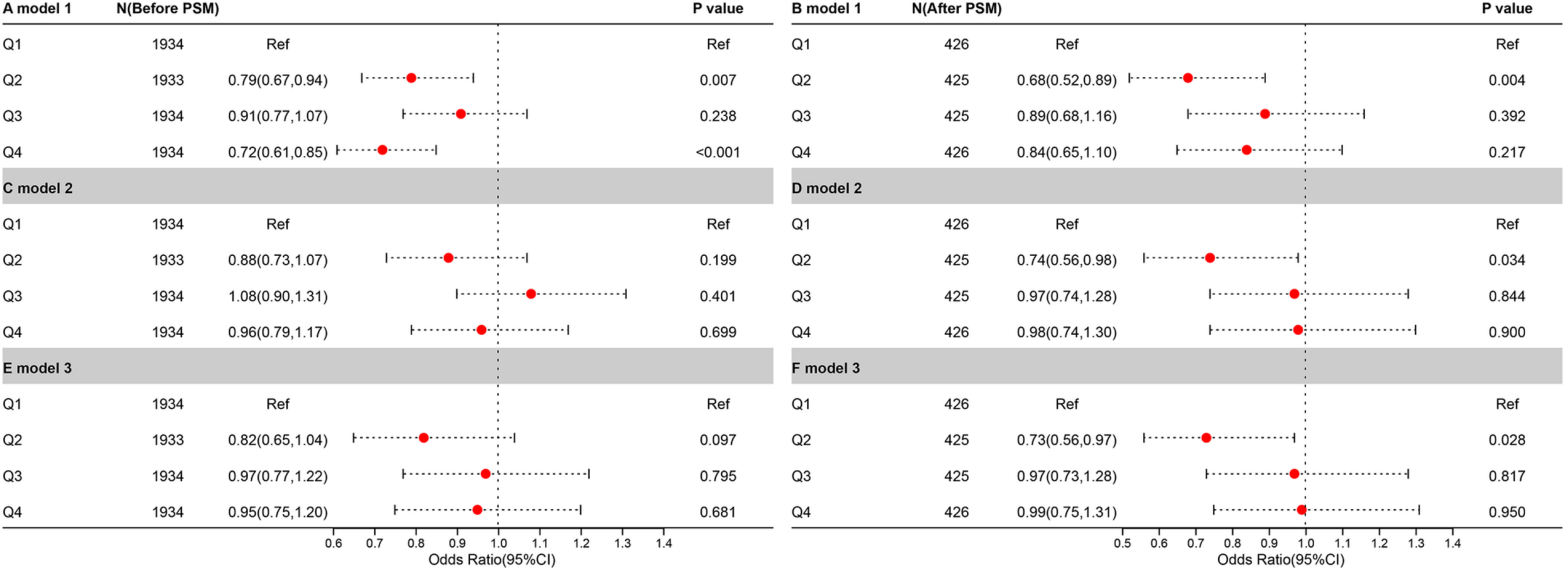
The relationship between UUI prevalence and CDAI before and after PSM. It is denoted by the letters “A,” “C,” and “E,” before PSM. Meanwhile, it is denoted by the letters “B,” “D,” and “F,” after PSM.

To further explore the relationship between CDAI and UUI, a weighted logistic regression analysis of the three models was conducted (Figure 3). The CDAI was converted (Q1-4; Q1 was used as a reference point) and examined by quaternity. In Model 1, no variables have been added. In Model 2, race, Age, education, and PIR adjustments were made. Model 3 was built using Model 2, modified to account for BMI, smoking, alcohol consumption, hypertensive disease, diabetes, total cholesterol, moderate activity, and vigorous activity.

In the pre-PSM analysis, only Q2 (*p* = 0.007) and Q4 (*p* < 0.001) were negatively correlated with UUI in the crude model, and this relationship was lost in the other models. In the analysis following the PSM, a negative correlation between Q2 and UUI was found in all three models (*p* = 0.004 in the crude model, *p* = 0.034 in model 2, and *p* = 0.028 in model 3).

### 3.4 Connectivity between CDAI and UUI in terms of dose–response

In the threshold effect analysis, we found no linear relationship between CDAI and UUI either before or after PSM (*p* = 0.383 and *p* = 0.483). Moreover, the negative correlation between CDAI greater than or less than the K value and UUI was not significant before PSM (*p* = 0.086 and *p* = 0.341). After PSM, CDAI was negatively correlated with UUI when CDAI was <K value (*p* = 0.027), and the relationship was not significant when it was > K value (Table 3).

**Table 3:** Relationship between CDAI and prevalence of UUI (analysis of the threshold effect).

Outcome	OR (95% CI), *p*	
	Prior to PSM	Following PSM
Model one		
Linear impact	0.99 (0.96, 1.02) 0.383	0.99 (0.97, 1.01) 0.483
Model two		
Inflection point (K)	0.951	0.523
CDAI < K	0.94 (0.88, 1.01) 0.086	0.91 (0.84, 0.99) 0.027
CDAI > K	0.99 (0.94, 1.05) 0.341	0.98 (0.94, 1.03) 0.265

This model included adjustments for all covariates.

**Figure 4:**
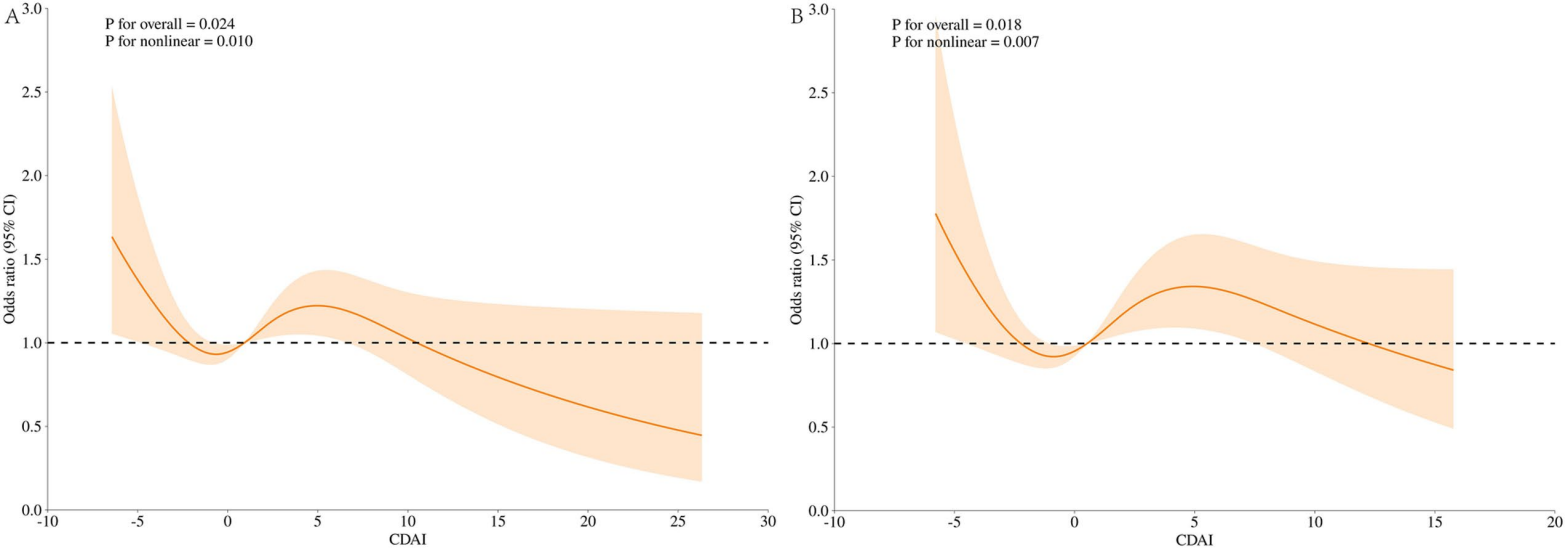
Dose–response analysis of UUI incidence (RCS). **(A)** Whole modified model, before PSM; **(B)** Whole modified model, after PSM. Terminologies: RCS, restricted cubic splines.

Figure 4 displays the results of the dose–response relationship for the Restricted Cubic Spline (RCS) (node setting of 4 before and after PSM). Under the fully adjusted model, CDAI and UUI prevalence showed a nonlinear relationship before and after PSM (nonlinear *p* of 0.010 before PSM, nonlinear *p* of 0.007 after PSM). At OR = 1, the reference values for CDAI before and after PSM were 0.776 and 0.523, respectively. The overall relationship between CDAI and UUI was significant (*p* for overall = 0.024 before PSM and *p* for overall = 0.018 after PSM).

### 3.5 Segmented regression analysis and sensitivity analysis

**Figure 5:**
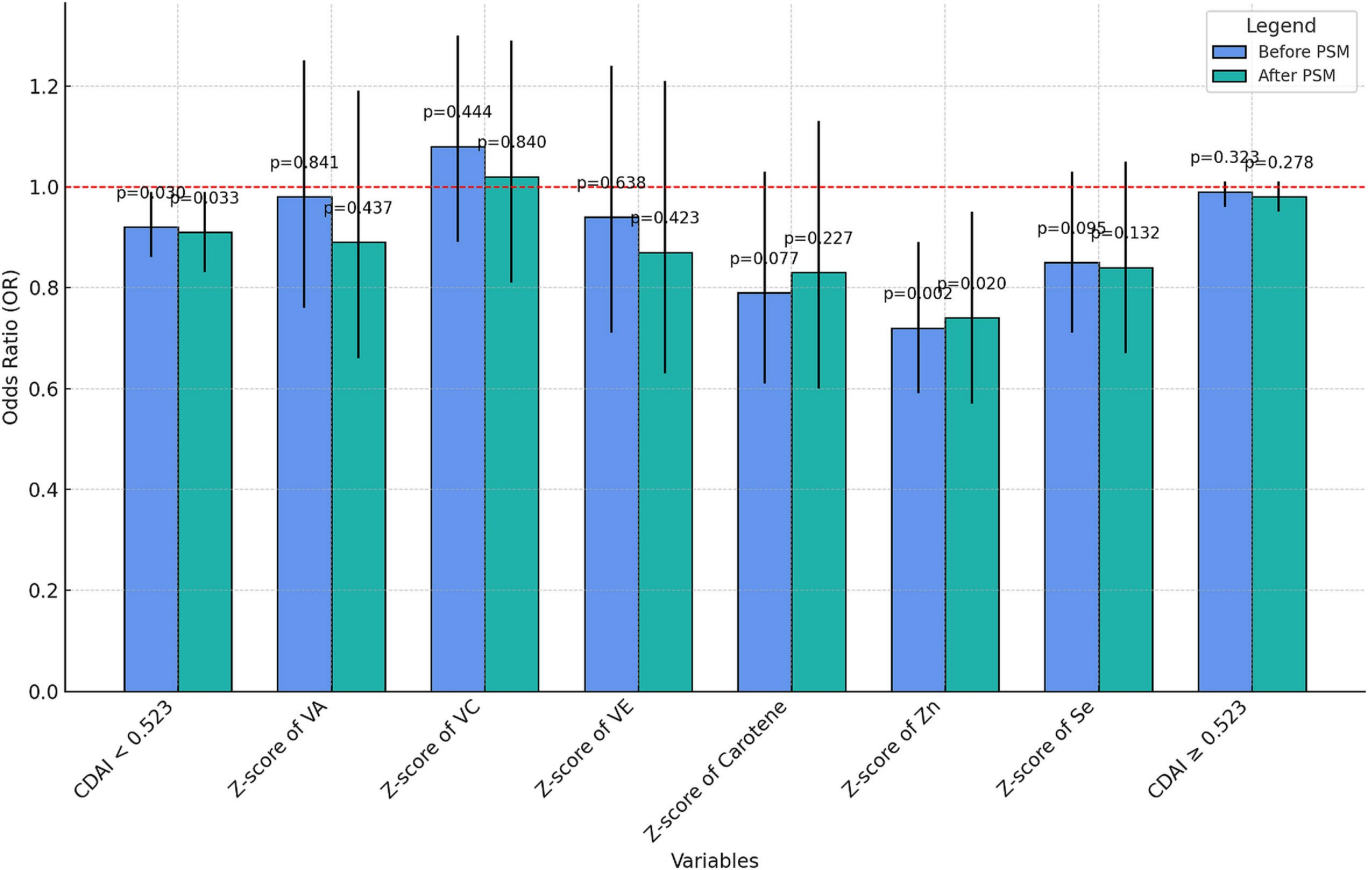
Grouped bar charts for regression analysis of CDAI and its components.

The K value after PSM (K = 0.523) was used to distinguish the two groups of people and the multiple regression analysis of CDAI and UUI was performed before and after PSM, respectively. The nutrient elements of each component in the CDAI<0.523 group were subjected to sensitivity analysis (Supplementary Table S2) and visualized (Figure 5).

The negative correlation with UUI was not significant for CDAI ≥0.523 either before or after PSM (*p* = 0.323 before PSM, *p* = 0.278 after PSM). However, at a CDAI less than 0.523, the CDAI was negatively correlated with UUI incidence both before and after PSM (OR = 0.92, *p* = 0.03 before PSM and OR = 0.91, *p* = 0.033 after PSM). Moreover, among the components, only the z-score of zinc showed a relatively significant negative correlation with UUI (OR = 0.72, *p* = 0.002 before PSM and OR = 0.74, *p* = 0.02 after PSM), and no significant correlation was found between the rest of the components and the incidence of UUI.

**Figure 6:**
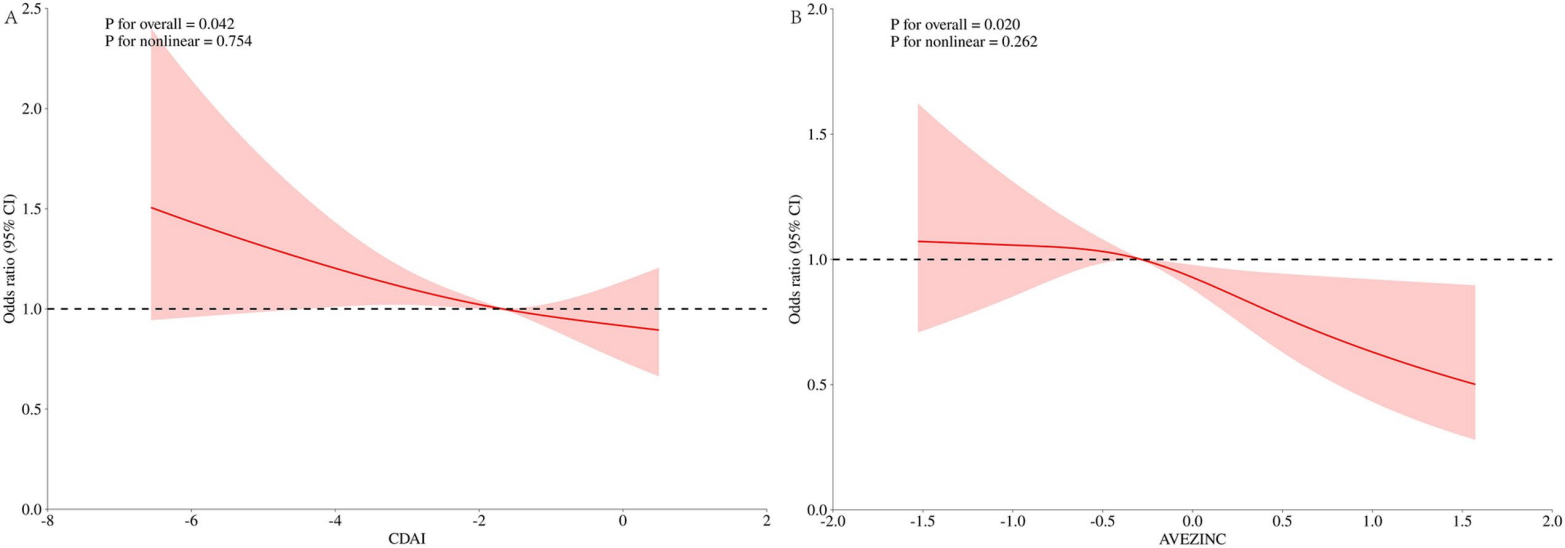
Dose–response analysis of UUI incidence (RCS). **(A)** Whole modified model, CDAI; **(B)** whole modified model, Zinc. Terminologies: RCS, restricted cubic splines.

To further explore the relationship between CDAI and zinc with UUI, we performed RCS plots for CDAI and zinc, respectively, under the fully adjusted model with the node setting of 3 before PSM (Figure 6). Neither the CDAI nor the z score of Zinc showed a nonlinear relationship with UUI (CDAI, nonlinear *p* of 0.754, Zinc, nonlinear *p* of 0.262). At OR = 1, the reference values were − 1.672 and − 0.287, respectively. The overall *p*-value for the RCS plotted for the relationship between CDAI and UUI was 0.042.

### 3.6 Subgroup analysis between CDAI and incidence of UUI

The study stratified the first subsection by race, age, education, PIR, body mass index, diabetes mellitus, hypertension, whether or not they smoked, whether or not they drank alcohol, total cholesterol, and moderate and vigorous activity (Table 4). Analyses were statistically significant regardless of pre- and post-PSM (*p* = 0.03 before PSM and *p* = 0.033 after PSM). Before and after PSM, CDAI was significantly negatively associated with UUI among those whose race was other Hispanic, higher than high school education, high PIR, non-drinkers, diabetics, and high cholesterol levels. In addition, after PSM, CDAI was negatively associated with UUI among those aged ≥50 years. Moreover, race played a significant moderating role in the effect after PSM, *p* for interaction = 0.043. It suggests a significant difference between racial subgroups. Apart from this, no significant interaction effects were found in other subgroups regardless of before or after PSM, suggesting that our results apply to almost all types of male populations.

**Table 4:** Stratified relationships between CDAI and UUI prevalence in U.S. male adults.

Subgroup	Before PSM			After PSM		
	OR (95%CI)	*p*	*p* for interaction	OR (95%CI)	*p*	*p* for interaction
All patients	0.92 (0.86 ~ 0.99)	0.030		0.91 (0.83 ~ 0.99)	0.033	
Age (years)			0.533			0.782
<50	0.89 (0.77 ~ 1.03)	0.111		0.94 (0.75 ~ 1.17)	0.564	
≥50	0.93 (0.86 ~ 1.01)	0.104		0.90 (0.81 ~ 0.99)	0.034	
Race			0.118			0.043
Mexican American	0.94 (0.76 ~ 1.17)	0.599		1.01 (0.78 ~ 1.32)	0.931	
Other Hispanic	0.73 (0.58 ~ 0.93)	0.011		0.67 (0.47 ~ 0.94)	0.022	
Non-Hispanic white	0.93 (0.82 ~ 1.05)	0.230		0.86 (0.73 ~ 1.00)	0.055	
Non-Hispanic black	0.92 (0.81 ~ 1.04)	0.168		0.89 (0.76 ~ 1.06)	0.193	
Other	1.27 (0.95 ~ 1.70)	0.107		1.39 (0.95 ~ 2.05)	0.089	
Education			0.581			0.475
Below high school level	0.93 (0.82 ~ 1.06)	0.291		0.90 (0.76 ~ 1.06)	0.211	
High school diploma	0.98 (0.85 ~ 1.14)	0.796		1.06 (0.87 ~ 1.28)	0.572	
More than high school	0.88 (0.79 ~ 0.98)	0.025		0.87 (0.76 ~ 1.00)	0.042	
PIR			0.617			0.638
<2	0.94 (0.86 ~ 1.03)	0.194		0.94 (0.83 ~ 1.06)	0.286	
≥2	0.87 (0.78 ~ 0.98)	0.025		0.86 (0.75 ~ 0.99)	0.039	
BMI (kg/m2)			0.268			0.908
<25	0.89 (0.76 ~ 1.03)	0.127		0.89 (0.73 ~ 1.08)	0.227	
25–29.99	0.99 (0.87 ~ 1.12)	0.854		0.92 (0.79 ~ 1.08)	0.314	
≥30	0.91 (0.81 ~ 1.01)	0.079		0.92 (0.80 ~ 1.06)	0.247	
Smoking			0.821			0.913
Yes	0.93 (0.85 ~ 1.02)	0.105		0.91 (0.82 ~ 1.01)	0.087	
No	0.91 (0.80 ~ 1.04)	0.165		0.91 (0.76 ~ 1.09)	0.304	
Alcohol consumption			0.648			0.806
No	0.92 (0.86 ~ 0.99)	0.027		0.91 (0.83 ~ 0.99)	0.033	
Yes	0.00 (0.00 ~ Inf)	0.998		0.00 (0.00 ~ Inf)	1.000	
Hypertension			0.935			0.751
No	0.90 (0.80 ~ 1.02)	0.093		0.88 (0.75 ~ 1.03)	0.117	
Yes	0.93 (0.85 ~ 1.02)	0.129		0.92 (0.82 ~ 1.03)	0.130	
Diabetes			0.517			0.447
No	0.94 (0.86 ~ 1.03)	0.203		0.94 (0.84 ~ 1.05)	0.284	
Yes	0.87 (0.76 ~ 0.98)	0.027		0.84 (0.72 ~ 0.98)	0.025	
Vigorous activity status			0.671			0.715
No	0.92 (0.85 ~ 1.00)	0.064		0.91 (0.81 ~ 1.01)	0.066	
Yes	0.93 (0.80 ~ 1.07)	0.298		0.94 (0.78 ~ 1.14)	0.545	
Moderate activity status			0.487			0.543
No	0.93 (0.85 ~ 1.02)	0.144		0.92 (0.81 ~ 1.03)	0.158	
Yes	0.91 (0.81 ~ 1.01)	0.077		0.88 (0.77 ~ 1.01)	0.073	
Total cholesterol			0.674			0.244
Low level	0.95 (0.86 ~ 1.06)	0.379		0.98 (0.86 ~ 1.13)	0.798	
High level	0.89 (0.81 ~ 0.99)	0.025		0.84 (0.75 ~ 0.95)	0.006	

All covariates have been adjusted without adjusting the stratified variable itself. OR, odds ratio; CI, confidence interval.

### 3.7 Investigating possible mechanistic connections

**Figure 7:**
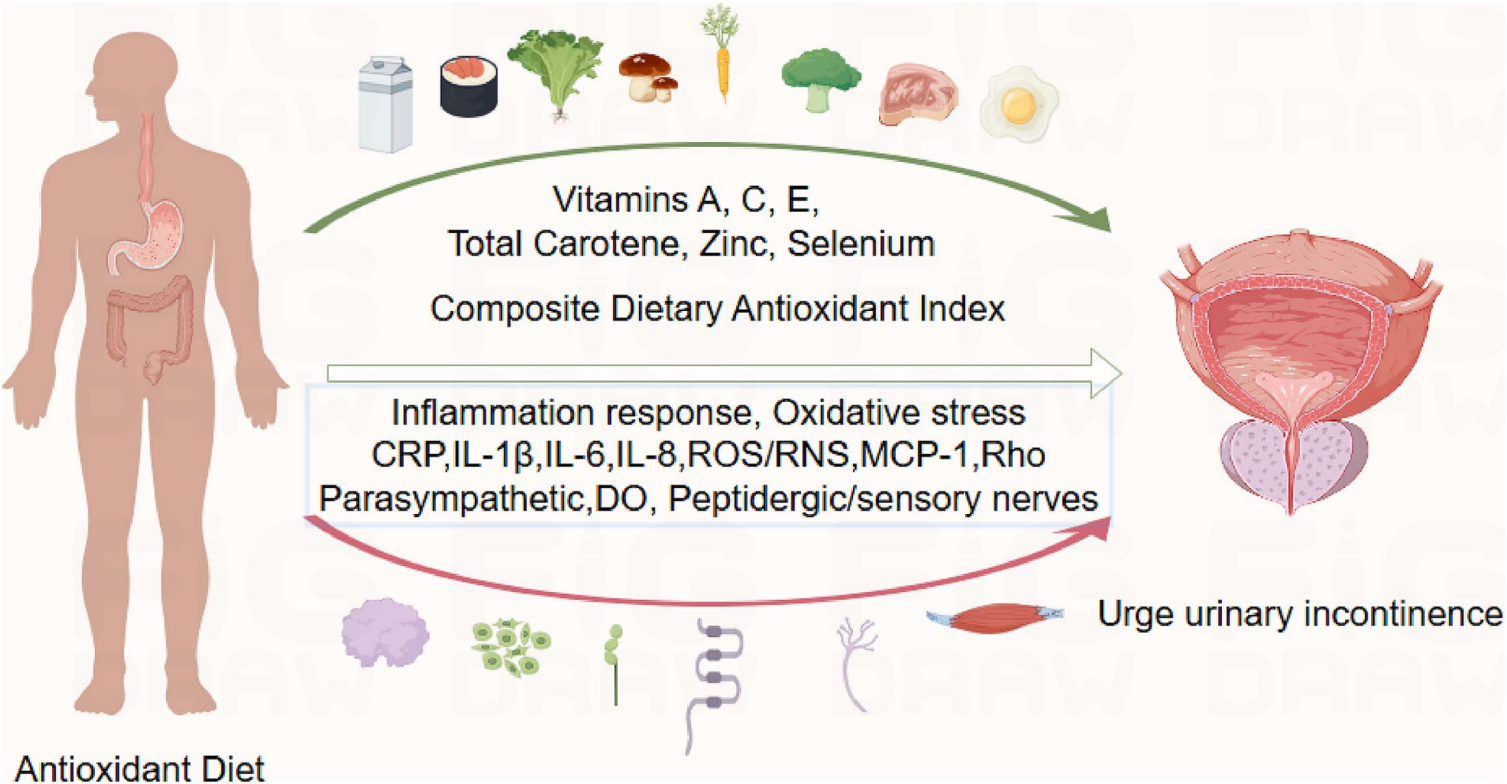
Intuitive diagram of CDAI and UUI analysis.

Based on Figdraw, an intuitive diagram is presented in Figure 7. We discuss the mechanisms by which CDAI affects the prevalence of UUI in Part 4. In the figure, the green arrows represent typical foods rich in the Composite Dietary Antioxidant Index (CDAI), which are beneficial for reducing the risk of UUI. The red arrows indicate biological mechanisms discussed in the article that contribute to the occurrence of UUI. These include markers of inflammation and oxidative stress: CRP (C-reactive protein), IL-1β, IL-6, IL-8 (cytokines), ROS/RNS (reactive oxygen and nitrogen species), and MCP-1 (monocyte chemoattractant protein-1). Other involved factors include Rho (Ras homologous), parasympathetic nerves, detrusor overactivity (DO), and peptidergic/sensory nerves (For more details, please refer to the discussion section).

## 4 Discussion

In this cross-sectional study of a nationally representative sample of U.S. men, greater consumption of antioxidant micronutrients was connected to a reduced likelihood of UUI in men with subthreshold CDAI intake (K = 0.523). However, the CDAI was not significantly associated with SUI or MUI.

Overactive bladder (OAB) syndrome is frequently accompanied by UUI. Prior research has indicated that 82.9% of patients with OAB have UUI (41). The connection between inflammation and overactive bladder (OAB) has been the subject of several investigations. Increased CRP levels were consistently linked to OAB in men, according to a study that investigated people in Boston between the ages of 30 and 79 (42). The idea that inflammatory infections may be an undervalued factor in the etiology of certain OAB patients is supported by a review article that discusses important findings from recent clinical and laboratory studies, including the relationship between bladder inflammation, urinary tract infections, and OAB pathogenesis (43).

Studies of the pathophysiology of urolithiasis have found that UUI involves molecular mechanisms: signalling and inflammation (44) (Figure 7). Increased serum C-reactive protein (CRP) levels in patients with UUI compared to non-patients have been demonstrated in several studies (45). Studies examining ischemia-modified albumin (IMA) have found that IMA levels are significantly elevated in patients with UUI compared to non-UUI patients (46). IMA levels are elevated in Systemic Inflammatory Response Syndrome (SIRS) and some inflammatory diseases. This may be related to the large number of free radicals released during the inflammatory process. Serum levels of interleukin-1β, −6 and − 8 have also been found to be elevated in UUI patients (47). IL-6 and IL-1β: elevated levels in a variety of chronic inflammatory diseases drive long-term inflammatory responses. IL-8 attracts primarily neutrophils to sites of inflammation and promotes infiltration of inflammatory cells. Urine levels of monocyte chemotactic protein 1 (MCP-1) in patients with UUI showed a trend toward higher levels compared to non-UUI patients (46). The pathophysiology of bladder dysfunction and the control of connexin expression are significantly influenced by inflammatory cytokines (48). Additionally, the relationship between local immune cells and overactive bladder parasympathetic and peptidergic/sensory innervation is mediated by inflammatory cytokines (49).

A “vicious cycle” can be created when oxidative stress triggers an inflammatory response by triggering inflammatory signalling pathways, which can further intensify oxidative stress. In an acute inflammatory response, neutrophils and macrophages kill pathogens by releasing large amounts of ROS through an oxidative burst (respiratory burst). The accumulation of reactive oxygen species (ROS) leads to the oxidation of DNA, proteins, carbohydrates lipids, and apoptosis (50). Diet controls the plasma redox state as an external factor and shields the body from reactive oxygen and reactive nitrogen species. Scavenging oxidants and antioxidants prevent oxidative damage by preserving a stable cellular redox state (51). Exogenous intake of antioxidants may prevent urinary incontinence and bladder ischemia (52, 53).

Our research revealed that CDAI was negatively and linearly correlated with UUI in men when CDAI was below 0.523 (median CDAI: 0.776). However, this relationship lost its significance when the CDAI value was greater than 0.523. This may be related to the saturating effect of dietary antioxidants. According to a study, dietary antioxidants and depression in persons who are overweight or obese are negatively correlated. However, in the group that was overweight, saturation effects were noted (54). In the study of dietary antioxidants and coronary heart disease, a threshold effect of complex dietary antioxidants was also found (55). A study examining dietary anti-inflammation and cognitive dysfunction in older adults also found a saturating effect of complex dietary antioxidants (56). In addition, excessive vitamin C intake is positively associated with the development of urinary incontinence (52). Because of the threshold effect, antioxidants have been shown to exhibit opposite effects under certain conditions (57).

More crucially, in the study of oxidative balance fractions and urinary incontinence, behavioural oxidative balance fractions were found to have a much greater effect on urinary incontinence than dietary oxidative balance fractions. And the behavioural oxidative balance score included physical activity, body mass index, alcohol consumption, and cotinine (58). All these four variables were included in our covariates, which had a significant impact on the prevalence of CDAI and UUI. As a result, at a CDAI greater than 0.523, the negative connection with UUI prevalence is no longer significant. In our stratified analyses, we found that the relationship between the prevalence of CDAI and UUI did not differ significantly in populations differing in BMI, smoking, and vigorous or moderate activity, regardless of before or after PSM. The relationship between the prevalence of UUI and CDAI was more significant only in those who did not drink alcohol.

We further stratified by each covariate and found no significant interaction effects before or after PSM except for the variable race after PSM, suggesting that our findings are applicable to almost all types of male populations. In subgroup analyses, there was greater sensitivity to the protective effects of CDAI among those whose race was other Hispanic, higher than high school education level, PIR ≥2, did not consume alcohol, had diabetes, and had high cholesterol levels. An article examining racial differences in overactive bladder found that urge incontinence has a high prevalence among Hispanics (59). Alcohol intake is positively associated with lower urinary tract disorders such as OAB (60). Several studies have confirmed that the prevalence of UI is increased in poor populations and exacerbated by complex social, cultural and psychological influences (61). It has been shown that the correlation between diabetes and OAB is more significant through systemic inflammation as a mediator (62). Research on the relationship between hypercholesterolemia and UUI is still lacking, but a study in rats found that hypercholesterolemia was positively associated with detrusor overactivity (DO) (63). In summary, we have identified specific beneficiary populations most likely to benefit from increased antioxidant dietary micronutrients to reduce the prevalence of UUI. In adult males with CDAI levels below 0.523, increasing dietary antioxidant intake is associated with a decrease in the incidence of UUI. This may provide some theoretical basis for the prevention or dietary treatment of UUI in clinical as well as public health settings.

In our compositional analysis, we found that the negative correlation between zinc and the incidence of UUI was more pronounced. The study found that zinc plays a significant role in antioxidant defense and inflammation regulation (64). Zinc acts as a cofactor for the antioxidant enzyme superoxide dismutase (SOD1), facilitating the conversion of superoxide radicals into less harmful molecules, thereby reducing oxidative stress and preventing cellular damage. In addition, zinc plays an important role in neural function by regulating neurotransmitter release, supporting synaptic plasticity, and protecting neurons from oxidative damage (65). Zinc regulates inflammation by modulating the production of pro-inflammatory cytokines (such as TNF-*α* and IL-6) and promoting an anti-inflammatory immune response, primarily through the inhibition of NF-κB activation (66). Therefore, zinc, as an important component of CDAI, plays a significant protective role in the risk of UUI. This study utilized a large, representative sample of adult males from NHANES and followed a carefully designed research protocol. To the best of our knowledge, this is the first study to explore the association between CDAI and UUI in men using a large sample size. We used univariate regression, multivariate regression, threshold effects analysis, sensitivity analysis, and subgroup analysis to enhance our understanding of their relationship. We investigated the relationship between composite dietary trace elements, which better reflect real-life scenarios, and the incidence of UUI in men. Additionally, we identified the threshold of CDAI that influences UUI in men. This threshold could be beneficial in clinical practice for preventing UUI and predicting the risk of UUI in adult men. Nevertheless, there are certain unavoidable limits to our study. First, since the study was cross-sectional, it was impossible for us to establish causality. Therefore, future longitudinal studies or randomized controlled trials are needed to better understand the causal relationship. Second, the potential bias easily introduced by dietary data from interviews leads to potentially biased results. Finally, we are unable to determine the residual confounding effects that may arise from unmeasured factors. Therefore, more research is needed in the future to address these limitations and provide further insights.

## 5 Conclusion

This study sought to increase understanding of the role antioxidant diets play in UUI prevalence among men. The results showed that when CDAI was below the threshold, the incidence of UUI was negatively correlated with CDAI. Therefore, CDAI can be used to predict the risk of UUI in men and guide the prevention of UUI in men. Men with lower dietary antioxidant micronutrient intake (approximately half of the adult male population in the United States) experience a reduced risk of UUI as their dietary antioxidant intake increases.

## Data Availability

The original contributions presented in the study are included in the article/Supplementary material, further inquiries can be directed to the corresponding authors.

## Footnotes

^1^ https://www.cdc.gov/nchs/nhanes
