# Supplementary material for: Composite dietary antioxidant index in relation to urge urinary incontinence in US men
Source: Front Nutr. 2024 Dec 20;11:1514320. doi: 10.3389/fnut.2024.1514320 (PMC11695336; doi:10.3389/fnut.2024.1514320)
Supplement: Supplementary file 1 [file Table_1.docx]

Supplementary table 1 Weighted basic characteristics of screened participants(N=1702) after PSM

| Characteristics | Total (n = 1702) | CDAI | | | | P-value |
| --- | --- | --- | --- | --- | --- | --- |
|  |  | Q1 (n = 426) | Q2 (n = 425) | Q3 (n = 425) | Q4 (n = 426) |  |
| Age, years |  |  |  |  |  | **0.025** |
| <50 | 328 (19.27) | 83 (19.48) | 70 (16.47) | 73 (17.18) | 102 (23.94) |  |
| ≥50 | 1374 (80.73) | 343 (80.52) | 355 (83.53) | 352 (82.82) | 324 (76.06) |  |
| Race |  |  |  |  |  | **<0.001** |
| Mexican American | 211 (12.40) | 50 (11.74) | 47 (11.06) | 52 (12.24) | 62 (14.55) |  |
| Other Hispanic | 157 (9.22) | 49 (11.50) | 41 (9.65) | 38 (8.94) | 29 (6.81) |  |
| Non-Hispanic white | 692 (40.66) | 140 (32.86) | 187 (44.00) | 183 (43.06) | 182 (42.72) |  |
| Non-Hispanic black | 451 (26.50) | 147 (34.51) | 101 (23.76) | 113 (26.59) | 90 (21.13) |  |
| Other | 191 (11.22) | 40 (9.39) | 49 (11.53) | 39 (9.18) | 63 (14.79) |  |
| Education |  |  |  |  |  | **<0.001** |
| Less than high school | 356 (20.92) | 133 (31.22) | 84 (19.76) | 77 (18.12) | 62 (14.55) |  |
| High school diploma | 404 (23.74) | 109 (25.59) | 101 (23.76) | 98 (23.06) | 96 (22.54) |  |
| More than high school | 942 (55.35) | 184 (43.19) | 240 (56.47) | 250 (58.82) | 268 (62.91) |  |
| PIR |  |  |  |  |  | **<0.001** |
| <2 | 839 (49.29) | 264 (61.97) | 205 (48.24) | 183 (43.06) | 187 (43.90) |  |
| ≥2 | 863 (50.71) | 162 (38.03) | 220 (51.76) | 242 (56.94) | 239 (56.10) |  |
| BMI (kg/m^2^) |  |  |  |  |  | 0.086 |
| <25 | 391 (22.97) | 108 (25.35) | 77 (18.12) | 92 (21.65) | 114 (26.76) |  |
| 25-29.99 | 628 (36.90) | 149 (34.98) | 168 (39.53) | 160 (37.65) | 151 (35.45) |  |
| ≥30 | 683 (40.13) | 169 (39.67) | 180 (42.35) | 173 (40.71) | 161 (37.79) |  |
| Smoking |  |  |  |  |  | 0.059 |
| Yes | 1133 (66.57) | 303 (71.13) | 279 (65.65) | 285 (67.06) | 266 (62.44) |  |
| No | 569 (33.43) | 123 (28.87) | 146 (34.35) | 140 (32.94) | 160 (37.56) |  |
| Alcohol consumption |  |  |  |  |  | 0.145 |
| No | 1659 (97.47) | 417 (97.89) | 415 (97.65) | 418 (98.35) | 409 (96.01) |  |
| Yes | 43 (2.53) | 9 (2.11) | 10 (2.35) | 7 (1.65) | 17 (3.99) |  |
| Hypertension |  |  |  |  |  | **0.034** |
| No | 620 (36.43) | 142 (33.33) | 140 (32.94) | 163 (38.35) | 175 (41.08) |  |
| Yes | 1082 (63.57) | 284 (66.67) | 285 (67.06) | 262 (61.65) | 251 (58.92) |  |
| Diabetes |  |  |  |  |  | 0.524 |
| No | 1139 (66.92) | 289 (67.84) | 272 (64.00) | 287 (67.53) | 291 (68.31) |  |
| Yes | 563 (33.08) | 137 (32.16) | 153 (36.00) | 138 (32.47) | 135 (31.69) |  |
| Total cholesterol |  |  |  |  |  | **0.019** |
| Low level | 710 (41.72) | 201 (47.18) | 160 (37.65) | 165 (38.82) | 184 (43.19) |  |
| High level | 992 (58.28) | 225 (52.82) | 265 (62.35) | 260 (61.18) | 242 (56.81) |  |
| Vigorous activity |  |  |  |  |  | **0.039** |
| No | 1265 (74.32) | 330 (77.46) | 320 (75.29) | 320 (75.29) | 295 (69.25) |  |
| Yes | 437 (25.68) | 96 (22.54) | 105 (24.71) | 105 (24.71) | 131 (30.75) |  |
| Moderate activity |  |  |  |  |  | 0.087 |
| No | 980 (57.58) | 260 (61.03) | 256 (60.24) | 231 (54.35) | 233 (54.69) |  |
| Yes | 722 (42.42) | 166 (38.97) | 169 (39.76) | 194 (45.65) | 193 (45.31) |  |
| SUI |  |  |  |  |  | 0.508 |
| No | 1524 (89.54) | 382 (89.67) | 388 (91.29) | 375 (88.24) | 379 (88.97) |  |
| Yes | 178 (10.46) | 44 (10.33) | 37 (8.71) | 50 (11.76) | 47 (11.03) |  |
| UUI |  |  |  |  |  | **0.036** |
| No | 851 (50.00) | 195 (45.77) | 236 (55.53) | 207 (48.71) | 213 (50.00) |  |
| Yes | 851 (50.00) | 231 (54.23) | 189 (44.47) | 218 (51.29) | 213 (50.00) |  |
| MUI |  |  |  |  |  | 0.177 |
| No | 1548 (90.95) | 385 (90.38) | 397 (93.41) | 379 (89.18) | 387 (90.85) |  |
| Yes | 154 (9.05) | 41 (9.62) | 28 (6.59) | 46 (10.82) | 39 (9.15) |  |

Data are shown as n (%).
