# Supplementary material for: Composite dietary antioxidant index in relation to urge urinary incontinence in US men
Source: Front Nutr. 2024 Dec 20;11:1514320. doi: 10.3389/fnut.2024.1514320 (PMC11695336; doi:10.3389/fnut.2024.1514320)
Supplement: Supplementary file 2 [file Table_2.docx]

Supplementary table 2. Full factor-adjusted regression analysis of CDAI and its components

|  | Before PSM | | After PSM | |
| --- | --- | --- | --- | --- |
|  | OR (95%CI) | *P value* | OR (95%CI) | *P value* |
| CDAI < 0.523 | 0.92 (0.86, 0.99) | **0.030** | 0.91 (0.83, 0.99) | **0.033** |
| Z-score of VA | 0.98 (0.76, 1.25) | 0.841 | 0.89 (0.66, 1.19) | 0.437 |
| Z-score of VC | 1.08 (0.89, 1.30) | 0.444 | 1.02 (0.81, 1.29) | 0.840 |
| Z-score of VE | 0.94 (0.71, 1.24) | 0.638 | 0.87 (0.63, 1.21) | 0.423 |
| Z-score of Carotene | 0.79 (0.61, 1.03) | 0.077 | 0.83 (0.60, 1.13) | 0.227 |
| Z-score of Zn | 0.72 (0.59, 0.89) | **0.002** | 0.74 (0.57, 0.95) | **0.020** |
| Z-score of Se | 0.85 (0.71, 1.03) | 0.095 | 0.84 (0.67, 1.05) | 0.132 |
| CDAI ≥ 0.523 | 0.99 (0.96, 1.01) | 0.323 | 0.98 (0.95, 1.01) | 0.278 |

Performed under the fully adjusted model
