# Supplementary figures and images for: Composite dietary antioxidant index in relation to urge urinary incontinence in US men
Source: Front Nutr. 2024 Dec 20;11:1514320. doi: 10.3389/fnut.2024.1514320 (PMC11695336; doi:10.3389/fnut.2024.1514320)

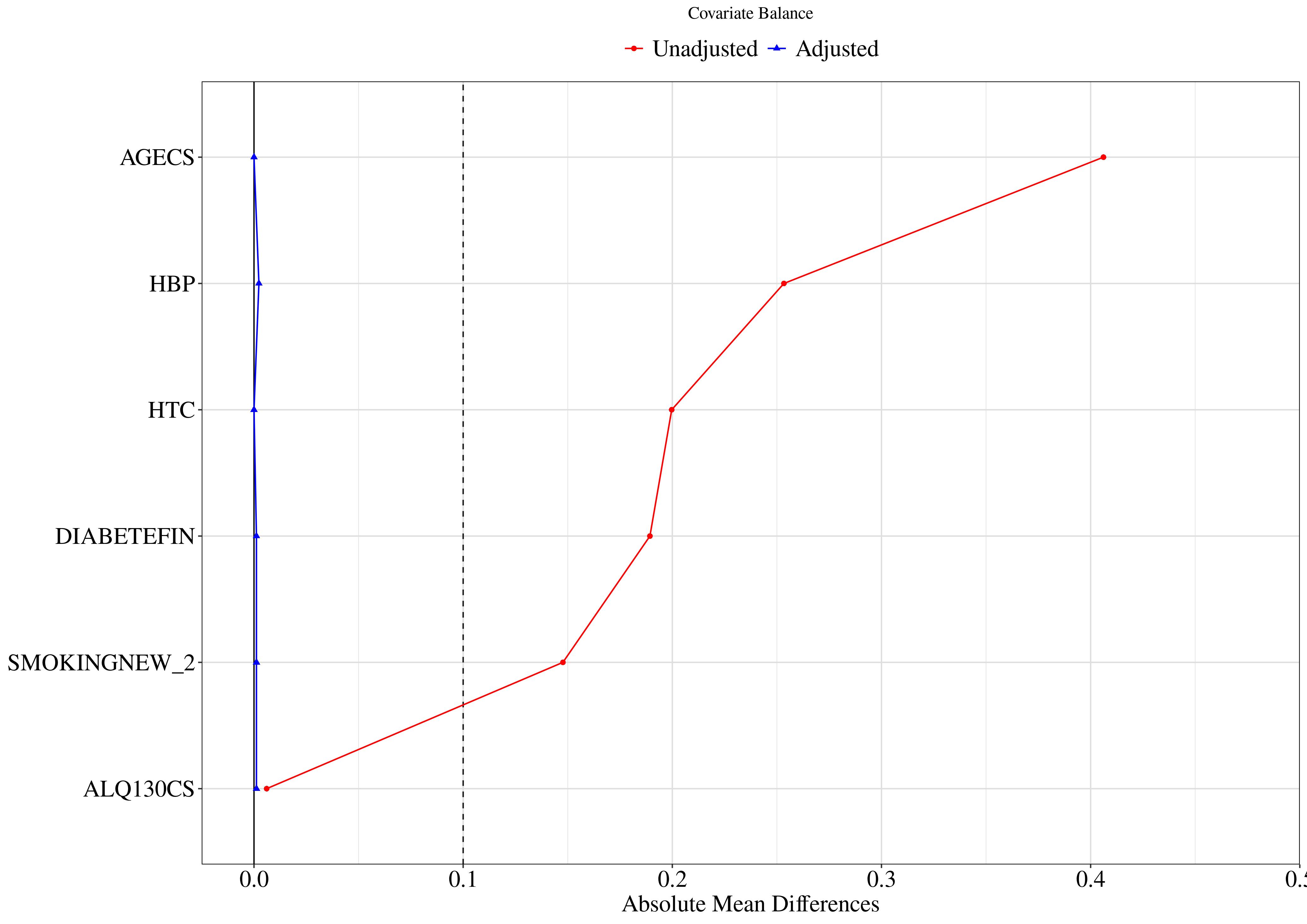

Supplement: Supplementary file 5 [file Image_1.tiff]

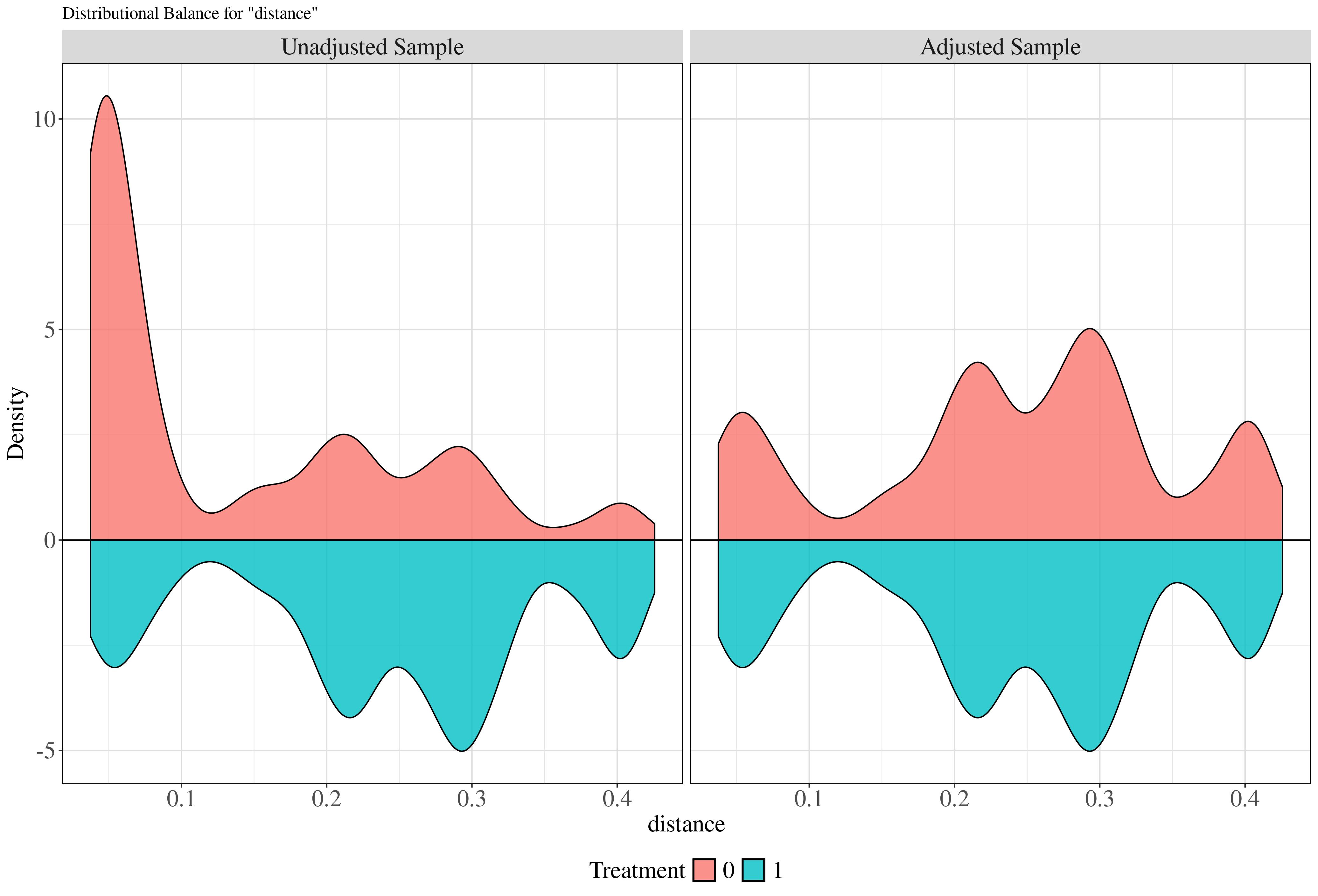

Supplement: Supplementary file 6 [file Image_2.tiff]
